# A Theory Based Intervention to Enhance Information Exchange during Over-The-Counter Consultations in Community Pharmacy: A Feasibility Study

**DOI:** 10.3390/pharmacy7020073

**Published:** 2019-06-20

**Authors:** Liza J. Seubert, Whitelaw Kerry, Hattingh Laetitia, Margaret C. Watson, Rhonda M. Clifford

**Affiliations:** 1 Division of Pharmacy, The University of Western Australia, M315, 35 Stirling Highway, Crawley WA 6009, Australia; kerry.whitelaw@uwa.edu.au (W.K.); rhonda.clifford@uwa.edu.au (R.M.C.); 2 School of Pharmacy and Pharmacology, Griffith University, Gold Coast Campus, Queensland 4222, Australia; l.hattingh@griffith.edu.au; 3 Gold Coast Health, Griffith University, Gold Coast, Queensland 4215, Australia; 4 Watson Research and Training Ltd., Aberdeen AB15 8FL, Scotland; magswatsonbusiness@gmail.com

**Keywords:** communication, nonprescription drugs, pharmacists, community pharmacy services, behaviour change, health behaviour, self care

## Abstract

**Background:** Management of minor ailments through self-care and self-medication brings both benefits and risks that can be mitigated if consumers and pharmacy personnel engage in information exchange during over-the-counter (OTC) consultations. **Objective:** Explore the feasibility of interventions using situational cues to promote information exchange between pharmacy personnel and consumers, during OTC consultations. **Methods:** Intervention tools were developed prior to conducting the study, in two community pharmacies in Perth, Western Australia. The situational cues included two posters and individual position badges. Data were collected from audio-recording OTC consultations, consumer questionnaires and interviews, and pharmacy personnel interviews. **Results:** Space required for posters and for researchers conducting interviews was challenging in the retail environment. Pharmacy personnel perceived that the badges positively impacted -consumers’ ability to identify the position of personnel they engaged with. Data collection methods were deemed practical and acceptable. **Conclusions:** The proposed interventions and evaluation methods were feasible. The use of posters and badges as situational cues to address the barriers to information exchange during OTC consultations was found to be practical, in a community pharmacy setting. There is potential to use situational cues to address other barriers identified to information exchange, to add to the effectiveness of the intervention. With growing emphasis on self-care and self-medication, effective interventions are necessary to promote information exchange to enhance appropriate management in community pharmacies.

## 1. Introduction

Consumers manage their minor ailments through self-care and self-medication [1,2]. The benefits of self-care and self-medication include convenience, and time and cost savings [2,3]. The potential risks include misdiagnosis, delaying appropriate treatment, interactions with concomitant medicines, and inappropriate use of medicines [2,4,5].

A wide range of over-the-counter (OTC) medicines is accessible via community pharmacies, and this is increasing globally [5,6,7,8]. To access these OTC medicines and health advice, consumers interact with community pharmacy personnel, during OTC consultations. A number of factors influence the effectiveness of OTC consultations, including the communication skills and clinical knowledge of pharmacy personnel, an appropriate level of pharmacist involvement, and the two-way exchange of relevant information between pharmacy personnel and consumers [9,10,11]. The level of interaction is also influenced by the legal requirements, which might vary with the jurisdiction or the country. In some jurisdictions, OTC medicines are available for consumer self-selection, who purchase them from community pharmacies without interacting with trained personnel [12,13,14,15]. In other jurisdictions, OTC medicines are regulated via classifications, where some OTC medicines are required to be kept out of the reach of consumers and only supplied once a pharmacist or other trained personnel has determined the clinical appropriateness of the medicine [16,17]. In Australia, OTC medicines are classified as unscheduled medicines, available from pharmacies or other retail outlets (e.g., supermarkets); Pharmacy Medicines are available only from community pharmacies; and Pharmacist Only Medicines are also only available from pharmacies under the supervision of a pharmacist [18,19]. A similar system exists in Canada [20] and New Zealand [21]. In the UK, OTC medicines are classified as Pharmacy Medicines (available only under the supervision of a pharmacist) and General Sales List medicines (available from pharmacies and other retail outlets) [16]. In the USA and many European countries, there are two classifications—prescription medicines are prescribed and then supplied through a pharmacy, and OTC medicines are available to general retail outlets, including pharmacies [22,23,24]. There is evidence that the management of OTC consultations is often suboptimal, primarily due to inadequate information gathering or inadequate information provision by pharmacy personnel [9,25,26,27,28]. Consumer resistance to information exchange has also been reported, as well as a lack of knowledge about the qualifications and responsibilities of pharmacists [29]. Investigation to understand and address these results is warranted.

The United Kingdom Medical Research Council recommends using theory to systematically develop complex interventions [30]. Interventions to enhance OTC consultations are complex, due to the variety of factors that influence this service, e.g., the type of enquiry (a specific product requested by name, advice about a symptom or condition) [11,25,31], clinical knowledge of the pharmacy personnel [9,31,32], communication skills of the pharmacy personnel [11], and privacy available for the consultation [11]. The Behaviour Change Wheel (BCW) is a validated methodological framework used to assist health researchers to apply the Capability, Opportunity, Motivation—Behaviour (COM–B) model of behaviour in any setting, to develop an intervention strategy [33]. The Theoretical Domains Framework (TDF), a validated derivation of the COM–B, allows for a more detailed understanding of the determinants of behaviour [34]. The BCW links intervention functions, which are the active components that can be used in an intervention strategy and can be observed and replicated, with behaviour change techniques (BCTs) [35].

This feasibility study is the fourth phase of a research programme that aims to enhance the quality of management of OTC consultations in community pharmacies:

Phase 1—Literature review [36].
Phase 2—Focus group discussions [29].
Phase 3—Intervention strategy development [37].
Phase 4—Intervention feasibility study.

In Phase 1, a systematic literature review identified interventions, to enhance communication during OTC consultations. Only five of the eleven identified studies were underpinned in theory, and all studies targeted pharmacy personnel [36]. There was minimal exploration of the consumer as a target of the intervention [36].

Barriers and facilitators for information exchange during OTC consultations were explored in the Phase 2 focus group discussions [29]. This phase was undertaken in Western Australia where most OTC medicines are located behind the counter and pharmacist involvement is dependent on the medicine’s legal classification [29]. In Western Australia, pharmacy assistants are required to complete training in the supply of OTC medicines [38], and pharmacists must be present for supervision, when the community pharmacy is open for trade [39]. The focus group discussions explored consumer and community pharmacy personnel perspectives about information exchange, and the results were coded and mapped to the TDF [29].

In Phase 3, using the BCW framework, intervention functions and the resulting BCTs that would most suitably address these barriers were identified [37]. Education, persuasion, environmental restructuring and modelling were determined to be potential intervention functions. The strategy developed that incorporated these functions was to display situational cues in the form of posters in a community pharmacy modelling information exchange behaviour, persuading consumers through highlighting the benefits of exchanging information, and educating consumers about its importance. Situational cues are defined as a “stimulus to action” [40,41,42,43] and can be environmental cues that provide important information or prompts to action.

**Figure 1:**
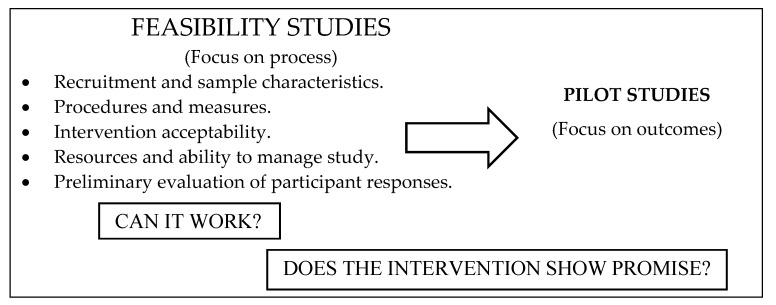
Distinctive features of a feasibility study [26].

The design of this feasibility study was based on the work of Bowen et al. [44] and the model described by Orsmond and Cohn [45] (Figure 1).

The aim of Phase 4 was to conduct a feasibility study to determine if the intervention strategy could work. The objectives of Phase 4 were:

Objective 1: Evaluate data collection procedures and outcome measures.
Objective 2: Preliminary exploration of the effect of posters on consumer perceptions about barriers to exchanging information.
Objective 3: Explore pharmacy personnel perceptions about OTC consultation behaviour during the intervention.
Objective 4: Evaluate the acceptability of the intervention study procedures to pharmacy personnel.

Secondary objective:

Objective 5: Evaluate poster interpretation with consumers.

## 2. Materials and Methods

Approval for conducting this study was obtained from the Human Research Ethics Committee at The University of Western Australia (RA/4/1/6538).

### 2.1. Intervention Strategy

The intervention strategy utilised situational cues in the form of posters depicting:

- A pharmacist with information about the qualifications and role of a pharmacist, and
- A consumer with an OTC enquiry engaging in information exchange highlighting the benefit of this behaviour and the reasons it is important.

An additional situational cue was developed in the form of an identity badge worn by pharmacy personnel, identifying their position as either a pharmacist or pharmacy assistant.

The posters were to be displayed in community pharmacies and the badges worn by pharmacy personnel. In a full-scale intervention study, the effects of each poster and the badges would be measured by implementing each separately, and collecting data on OTC consultations. Table 1 describes the stages of the feasibility study.

**Figure 2:**
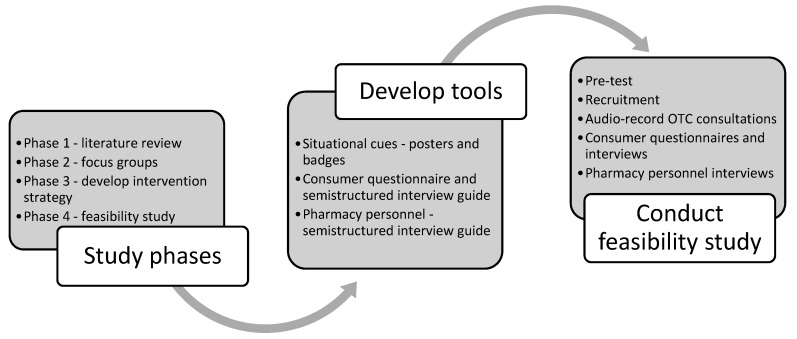
Study process.

Prior to conducting the study, a number of tools were developed and validity testing was conducted. Tools were also developed for the collection of data, to test the feasibility of the intervention (Figure 2).

### 2.2. Situational Cue Development

Phases 2 and 3 of the research [29,37] identified the barriers to information exchange during OTC consultations. Consumers were unaware that it is a pharmacist’s professional role and responsibility to gather information from consumers about their enquiry, to enable the former to provide appropriate advice. Consumers also found it difficult to differentiate between the pharmacist and pharmacy assistant personnel. The ‘professional role’ barrier was addressed in two forms of situational cues—first, through a poster that focused on the role, responsibilities and qualifications of a pharmacist explaining that asking questions was to ‘ensure your best health’ (ProfRole poster); and second, through badges developed to denote the role of the pharmacist or the pharmacy assistant. A second poster addressed consumers’ beliefs about their own ‘lay’ capability to self-diagnose and self-select appropriate OTC medicines. The InfoExchange poster modelled information exchange between a pharmacist and a consumer, highlighting that pharmacists ask questions ‘for your safety’.

#### 2.2.1. Poster Development

Eight posters were drafted by two Master of Pharmacy students and one of the authors (L.J.S.), to include a variety of BCTs [35] to address the barriers to information exchange identified in Phases 2 and 3 [29,37]. The BCTs used in this study were—education (about the role and responsibilities of pharmacists, and information about health consequences); persuasion (about the value of providing health information) and modelling (how to engage in an OTC consultation).

#### 2.2.2. Testing of Posters

Academics experienced in pharmacy practice (n = 5) and final year Master of Pharmacy students (n = 10) reviewed the eight draft posters, to establish if the intended messages were received by the person viewing the poster. The participants were asked to describe the message the poster delivered to them. The messages described from five of the eight drafts, aligned with the intent, while messages from the other three were poorly interpreted. The effective components were consolidated into three posters which were then tested with consenting consumers (n = 10) in a community pharmacy. The messages were well-understood by consumer participants and the content was further refined and consolidated into two posters to avoid duplication. A graphic designer assisted with the design of the final posters.

The first poster provided information regarding the role, responsibility, and qualifications of a pharmacist (ProfRole poster), and that pharmacists asked questions to ‘ensure your best health’. It was designed to educate consumers about the role, responsibility, and qualifications of a pharmacist, and to persuade consumers to exchange information during OTC consultations. The second (InfoExchange poster) modelled information exchange behaviour using an example of an OTC consultation, to persuade consumers to engage with the pharmacist ‘for your safety’. It also addressed consumer beliefs about their own capabilities regarding understanding their own health, through text in speech bubbles. The intent was for the posters to be easy to read from different areas of a pharmacy, therefore 83.5 cm × 210 cm banner-style posters were produced (Supplementary Material 1).

#### 2.2.3. Badge Development

The professional role of the pharmacist was also addressed through the development of badges to assist with identification. Badges printed with “PHARMACIST” or “PHARMACY ASSISTANT” were produced with a large font size ‘Source Sans Pro Semibold’ at 32 point, i.e., much larger than that typically usually used on badges. The badges had black text on white background with no other information or logos to enable consumers to quickly and easily identify if they were interacting with a pharmacist or assistant (Supplementary Material 2).

### 2.3. Pre-Test of Intervention Study Procedures

To inform the processes required for the study, a pre-test of pharmacy personnel recruitment, OTC consultation audio-recordings, and consumer questionnaire recruitment was conducted in a community pharmacy, before the feasibility study. The Critical Path Method [46] and systems thinking [47] were used to develop resources and procedures for the pre-test. Data were collected between 9 am and 3 pm on two weekdays (Tuesday and Friday) in September 2015. A logbook was used for researchers to record the field notes.

Audio-recordings of OTC consultations between pharmacy personnel and consumers were obtained by the pharmacy personnel who were wearing Philips Voice Tracer DVT500 digital recorders on a lanyard around their necks, with or without a lapel microphone. The purpose of the audio-recordings was to obtain authentic information about OTC consultations, which could serve as a data source for potential changes at the different stages of the intervention.

Researchers also approached consumers to participate in a questionnaire as they were leaving the pharmacy. A recruitment rate of 32% was recorded.

Posters and badges were not pre-tested. The pre-test informed the following processes:

(a) Recruitment of pharmacy personnel.
(b) Recruitment of consumer participants.
(c) Logistics of audio-recording OTC consultations including optimal recorder settings.
(d) The number of hours of audio-recording to capture at least 50 OTC consultations.

### 2.4. Intervention Outcomes

*Objective 1: Evaluate data collection procedures and outcome measures.*

#### 2.4.1. Audio-Recording OTC Consultations

Researchers set the recorders to the optimal settings, as per the pre-test, and provided them to pharmacy personnel participants. During the shift, researchers checked the devices to ensure they were recording, the settings had not changed, and that the battery was charged. At the end of the day, the recordings were downloaded and the devices were charged. The recordings were checked for audio quality.

#### 2.4.2. Consumer Questionnaire

A questionnaire was developed to quantitatively measure consumers’ perspectives about the identified barriers to information exchange during OTC consultations. The questionnaire was designed to determine changes in consumer perspectives over time, in the full-scale study. 

A validated generic TDF questionnaire developed by Huijg et al. was adapted for the purposes of this study [48]. Three items for each TDF domain containing the identified barriers (excepting the Environmental Context and Resources domain) were drafted, resulting in 16 questions. A 7-point Likert scale was used with the options ’strongly disagree’ to ‘strongly agree’, for 14 questions. The response to one question was described as ‘difficult’ to ‘easy’ and another was described as ‘not at all strong’ to ‘very strong’. Content validity of the draft items was assessed by a health psychologist and three experienced pharmacy academics, who provided feedback to refine the items prior to testing the questionnaire with a convenience sample of five consumers. Consumer participants were asked to comment on the face validity and fitness-for-purpose of each questionnaire item. Feedback was collated and the items were further refined, prior to randomization of the order of questions (Supplementary Material 3).

During week two of the feasibility study, consumers leaving the participating pharmacy were approached by researchers and asked to participate in the study. Written informed consent was obtained and the consumers completed the questionnaire.

#### 2.4.3. Evaluation of Posters

*Objective 5: Evaluate poster interpretation with consumers (secondary objective).*

The feasibility study provided an opportunity to further evaluate the interpretation of poster messages with a larger number of consumers. Consumers who participated with the questionnaire in 2.4.2 were asked to view an A4-sized copy of one of the posters. A semi-structured interview guide with prompts (Supplementary Material 4), consisting of questions to explore the consumer participants’ interpretation of and response to the posters, was developed. Consumers viewed one of the two posters and were then interviewed by a research assistant who took field notes during the interview.

*Objective 2: Preliminary exploration of effect of posters on consumer perceptions about barriers to exchanging information.*

Having viewed and discussed the poster, the consumer participants subsequently repeated the questionnaire.

### 2.5. Pharmacy Personnel Interviews

*Objective 3: Explore pharmacy personnel perceptions about OTC consultation behaviour during the intervention.*

and

*Objective 4: Evaluate the acceptability of the intervention study procedures to pharmacy personnel.*

A semi-structured interview guide was developed to explore the perceptions of pharmacy personnel regarding the feasibility of the intervention processes and the impact of the intervention on the information exchange. Practical issues about the use of situational cues in community pharmacy, wearing the audio-recorder, and research processes were also explored. One registered pharmacist and one intern pharmacist provided feedback on the draft interview questions, which were subsequently refined, and the final version contained three key questions and further prompt questions (Supplementary Material 4).

Semi-structured interviews were conducted with participant pharmacy personnel of the two community pharmacies during the four weeks following the intervention. The interviews were audio-recorded and the interviewer (L.J.S.) took field notes during each interview.

### 2.6. Participants

A convenience sample of two community pharmacies in metropolitan Perth with different business models was recruited. One was a traditional community pharmacy that used a hierarchical management style—reporting lines moved from junior to senior personnel, through to the pharmacist proprietor. The other was a contemporary community pharmacy with horizontal management, whereby all staff were encouraged to participate in decision-making. Both pharmacies were independent of marketing or franchise agreements and focused on the delivery of high quality services. Both pharmacies were located in affluent suburbs [49].

Proprietors of the pharmacies were approached to participate in the study and both consented. Informed consent and demographic information was obtained from pharmacy personnel. Whilst pharmacy participants were aware that the study focussed on OTC consultations, they were not informed about the focus on information exchange. In both pharmacies, personnel already wore different shirt colours to distinguish between pharmacist and pharmacy assistant. The personnel in one of the pharmacies also wore non-study badges with their first name (font size 16 point) and position (12 point) printed, accompanied by the pharmacy logo.

Consumer participants were recruited from both pharmacies. As consumers were leaving the two pharmacies, they were asked to participate in the study, and were provided a participant information sheet after which written consent was obtained.

### 2.7. Fidelity of the Study

Five research assistants were trained in the study methodology. Two were present in each pharmacy for six hours, from the beginning of trade on each Monday, Tuesday and Wednesday, over the first four weeks of the study. The research assistants provided the badges for pharmacy personnel to wear, they set up the posters in the pharmacies, and also recruited consumers to participate in the interview and complete the questionnaires. They also facilitated audio-recordings of OTC consultations—these data will be reported elsewhere.

### 2.8. Feasibility Study

The study was conducted in the two community pharmacies over an eight weeks period in February and March 2016, with follow-up pharmacy personnel interviews in March and April 2016. The stages of the intervention are described in Table 1.

### 2.9. Data Handling and Analysis

Data from consumer questionnaires were entered into Excel, for analysis. Data were excluded if participants responded with words instead of using the Likert scale. If two numbers on the Likert scale were circled for the same question, it was treated as missing data. Descriptive statistics were used to summarise the demographic characteristics. The mean change in consumer responses was calculated using the questionnaire data.

Poster validation was conducted by evaluating data from the field notes, which were taken during the consumer interviews for evaluating the intended messages of the posters.

Pharmacy personnel interviews were transcribed verbatim. Two researchers (L.J.S., W.K.) independently read and re-read the transcriptions, prior to discussing the emerging themes and reaching a consensus.

## 3. Results

### 3.1. Intervention Outcomes

*Objective 1: Evaluate data collection procedures and outcome measures.*

#### 3.1.1. Audio-Recording OTC Consultations

The pre-test recorder settings produced recordings that were audible and suitable for transcription. The devices remained on the settings programmed by the researchers and the battery life was more than sufficient for a day of recording, without a re-charge.

#### 3.1.2. Consumer Questionnaires

The recruitment target was a minimum of 30 consumer participants at each pharmacy. Of the 77 completed questionnaires, 75 were completed correctly. Demographic information about participants is presented in Table 2. Participant age ranged from 18–99 years of age and all spoke English at home.

*Objective 2: Preliminary exploration of the effect of posters on consumer perceptions about barriers to exchanging information.*

Consumer questionnaire responses pre- and post-review of posters are presented in Table 3. This feasibility study did not have enough statistical power to reach statistical significance and, therefore, only descriptive statistics are presented.

*Objective 5: Evaluate poster interpretation with consumers (secondary objective).*

#### 3.1.3. Evaluation of Posters

Of the 77 semi-structured consumer interviews, 75 provided information that was usable. Consumers reported the main message they received from the posters was that the pharmacist would ask questions and that consumers should also ask questions. Consumers also emphasised the role of the pharmacist and that you can trust your pharmacist. The feedback was consistent with the intent of the posters. Data are presented in Supplementary Material 5.

### 3.2. Pharmacy Personnel Interviews

Interviews were conducted with 13 of the 19 pharmacy personnel participants. One interview could not be transcribed due to malfunction of the recording device and was excluded from the analyses, therefore, data were available for only 12 interviews. Six personnel who had participated in the study were on leave or were no longer employed at the pharmacy, when the interviews were conducted. The median (IQR) duration of these interviews was 7 (6–10) min in pharmacy 1 and 9 (8–12) min in pharmacy 2.

#### 3.2.1. Pharmacy Personnel Demographics

Of the 12 interviewees, the median (IQR) age was 39.5 (28.5–51.3) years, and they had about 14.0 (8.0–28.0) years of work experience in a pharmacy (Table 4).

#### 3.2.2. Pharmacy Personnel Perceptions about Changes during the Intervention

*Objective 3: Explore pharmacy personnel perceptions about OTC consultation behaviour during the intervention.*

The interview commenced with an open question about the participant’s past experiences with OTC consultations, to make the participant comfortable, and to indicate a focus on the OTC interactions. Then, participants were asked a broad, non-leading question, to ascertain if changes in OTC interactions had been noticed during the research period. Statements about changes during the intervention were made by some pharmacy assistants. They stated consumers took notice of the posters and badges or made general comments about them.

“Sometimes when they [consumers] saw the badges they asked what that was all about and you’d explain.”

Pharmacy Assistant 1, (hereafter, Pharmacy Assistant quotes are labelled by the letters ‘PA’ followed by the participant serial number, e.g., ‘1’)

“That something different was going on, people were looking at the poster and then walking in and noticed things were a little bit different.”

PA6

One pharmacy assistant commented on consumers’ ability to identify the position of pharmacy personnel when wearing the badge.

“When they [consumers] noticed me wearing the badge [they] would walk straight up to me and say ‘I’d like to speak to a pharmacist.’”

PA6

“The badges were a good size. It delineated between who was who. I think it says to the customer: ‘Look, I’m the pharmacy assistant. I’ve got knowledge. I’m not just a check-out chick’.”

PA2

Other pharmacy assistants did not report any changes relating to the intervention.

Several pharmacists noted that the badges were noticed by consumers due to the size of the font.

“I think because on our badges they’re really small but on yours they were really visible. So I think the pharmacist badges got a lot of notice.”

Pharmacist 1 (hereafter, Pharmacist quotes are labelled by the letter ‘P’ followed by the participant serial number, e.g., ‘1’)

Pharmacists stated that consumers were able to distinguish between the pharmacist and the pharmacy assistant.

“I actually noticed a lot of people looking at the pharmacist badge so when you go out to help someone they know straight away they’re speaking to the pharmacist so it just makes it an easier conversation ‘coz they know what level you’re at.”

P1

“You could see them [consumers] reading them [badges] maybe just for the recognition ‘Oh I am talking to the pharmacist’.”

P3

#### 3.2.3. Feasibility of the Research Process

*Objective 4: Evaluate the acceptability of the intervention study procedures to the pharmacy personnel.*

To evaluate the feasibility of the research process, pharmacy personnel were asked specific questions about having research conducted in their workplace.

##### How Do You Feel About Having Research and Researchers in the Pharmacy?

Participants expressed neutral to positive comments about being involved in research. Overall, they were interested in the research and appreciated being involved in a study addressing communication during OTC consultations between pharmacy personnel and consumers, as they felt it was a critical aspect of their everyday work.

“It’s good. I think anything you can do to improve is a good thing.”

P5

##### What Are Your Thoughts on Wearing the Recorder?

Some personnel were initially concerned about the consumers’ reaction to being recorded. They expected to be questioned about privacy or thought it would be intrusive to consumers. In general, however, they were surprised that consumers did not express concern about being recorded. Other comments were about their personal feelings about non work-related conversations being recorded.

“It kind of felt, like, impersonal, a little bit intrusional when we had to wear them when we were having our personal conversations.”

(PA6)

Several pharmacists were not concerned about wearing a recorder at all. Comments were made regarding the heaviness of the recorder and that it banged on to the boxes and counters.

##### Do You Feel It Altered the Way You Work?

Pharmacy personnel said either they were too busy for it to have any effect or that it did have an initial effect, but they soon became accustomed to it.

“The first couple of times I was probably more conscious about how I spoke then after a while I got used to it [recording device]. It was just there and that was it.”

(PA2)

##### What Worked Well with the Research Process? Did You Encounter any Problems with the Research Process?

Positive comments were made about the posters and the helpfulness of the research assistants conducting the questionnaire with consumers.

Problems encountered were related to the limited space available in the pharmacies, as the research assistants who were present three days each week needed some space. This meant that they sometimes occupied areas that pharmacy personnel might require access to e.g., counselling area or staff room.

With regards to space available in the pharmacy, a comment was also made about the size of the posters, which were 835 mm × 2100 mm.

“The posters were a little big for our pharmacy because we’re so crowded. But that’s just because we are small. I think if they’re [the posters] too small people wouldn’t notice them so there needs to be a balance.”

(P2)

##### What Would You Recommend to Improve the Process?

When asked if they could think of anything to improve the research process, comments were made regarding the recording device, such as making it smaller or using a lapel microphone.

During this interview process it was apparent that one pharmacy assistant did not receive the entire induction to the research, this affected her experience with the process.

“I think the only thing I would say is right at the start I didn’t feel I knew anything. It would probably be good to have known what was being done at the shop. It’s hard to get fully involved if you don’t really know what things are up, is it to do with me or not? Am I a member of this or I am not?”

(PA5)

## 4. Discussion

This study explored the feasibility of an intervention study utilising situational cues to enhance information exchange during OTC consultations in community pharmacies, using the model described by Orsmond and Cohn [45]. Interventions that use cues to stimulate behaviour change are reported to more likely be effective if research to understand the specifics of the situation are conducted first, and the intervention is tailored to suit these findings [50]. The situational cues in this study were informed by Phases 1–3 and used validated processes, based on behaviour change theory, to develop the content.

### 4.1. Objective 1: Evaluate Data Collection Procedures and Outcome Measures

This study demonstrated that the intervention study design as proposed was feasible. The feasibility study verified that data collection through audio-recording OTC consultations and consumer questionnaires was practical. The quality of the audio-recordings was sufficient for the recordings to be transcribed. Researchers were able to exceed the consumer questionnaire recruitment target, and over 97% of the questionnaires were completed correctly, indicating that it was well-understood. 

### 4.2. Objective 2: Preliminary Exploration of the Effect of Posters on Consumer Perceptions about Barriers to Exchanging Information

Consumer questionnaire responses before viewing a poster generally showed high Likert scores. This might have been influenced by the characteristics of consumers from the two community pharmacies. Both pharmacies were located in affluent areas and half the consumer participants had a tertiary qualification, which was considerably higher than the 23% with a tertiary qualification reported in the 2016 census [51]. All participants spoke English at home, compared to 74% reported in the census [51]. Additionally, the majority indicated that this was their regular pharmacy, which might have resulted in familiarity with pharmacy personnel and their role. Therefore, preliminary results of the effect of the posters on consumer perceptions were inconclusive. However, both posters had a small positive impact on consumers’ perceptions of the pharmacist’s role and responsibility, as for questionnaire statement 5—“It is a pharmacist’s job and responsibility to ask me questions about my health”—there was more agreement after viewing either poster. Acknowledgement of the pharmacist’s responsibility to ask questions might facilitate consumer engagement with information provision and exchange [52]. It should be noted that immediate recall of the consumers was tested. Administering the questionnaire after three or six months would provide information on the long-term effects of the posters.

### 4.3. Objective 5: Evaluate Poster Interpretation with Consumers (secondary objective)

Consumer interviews confirmed that the messages the consumers were intended to receive from the posters, were consistent with their interpretation of the messages. This further confirms the trustworthiness of the poster content [53].

### 4.4. Objective 3: Explore Pharmacy Personnel Perceptions about OTC Consultation Behaviour during the Intervention

A dominant theme expressed in post-study interviews with pharmacy personnel was that consumers noticed the badges. It was noted that consumers were much more forthcoming in asking to speak to the pharmacist if they were interacting with a pharmacy assistant wearing an intervention badge. This was despite the fact that in both pharmacies, the pharmacists wore a shirt of a different colour from that of the pharmacy assistants, and in one pharmacy, the personnel wore badges displaying their name, position and a pharmacy logo. Pharmacy personnel commented that the badges were a good size compared to the badges they already had. The perception was that consumers appreciated instantly knowing the position of the person they were talking to. It allowed them to make a decision about whether they thought the enquiry could be managed by a pharmacy assistant or if they needed to ask to speak with the pharmacist. This finding is supported by a 2014 Australian study exploring consumer awareness of the role of community pharmacists in health service provision, which found that consumers differentiated between pharmacist and non-pharmacist personnel roles [54]. Participants commented that pharmacists were able to provide information and ensure the safe use of medicines, whereas non-pharmacist personnel simply assisted with purchases. This very simple identification technique could easily be implemented by making the text larger on the pharmacy personnel badges that are already worn. It should be noted that in some jurisdictions it is not mandatory for pharmacists to be on duty when community pharmacies are open. This might limit the usefulness of knowing the position of the person the consumer is interacting with, as there may not be another option.

### 4.5. Objective 4: Evaluate the Acceptability of the Intervention Study Procedures to Pharmacy Personnel

Pharmacy personnel made suggestions to improve the implementation and practicality of the methodology. It was highlighted in one interview that a pharmacy assistant participant did not receive the full introduction to the study as planned and this affected her engagement with the process. Another procedural element highlighted in the interviews was that pharmacy personnel found the recorders heavy over time and that lapel microphones and being able to put the recorder in a pocket would be helpful. This option was available to them, however, the information clearly had not been relayed to participants. Ensuring that procedural aspects of an intervention are adhered to, in an environment that is busy and has pharmacy personnel working different shifts, is essential for the rigor of the intervention.

The space requirements for the intervention must also be considered. Pharmacy personnel commented that there was limited space availability in their pharmacy, to properly fit the large posters. The current economic climate places pressure on pharmacy owners to ensure retail space is used profitably [55]. The financial implications of using limited retail space for non-retail purposes needs to be balanced with the professional obligations of pharmacists. The size of the posters could be adjusted to suit the space available in the pharmacy—or only one poster could be displayed at a time rather than both being displayed concurrently. Additionally, the space required for the two researchers to collect data and not get in the way of the day-to-day activities was found to be challenging in a retail environment, and needs to be considered in future studies.

### 4.6. Strengths and Limitations

Qualitative and quantitative methods were used to gain an in-depth understanding of the feasibility of the interventions. The outcome measures were designed and pre-tested to provide reproducible processes for future studies. Both pharmacies were located in affluent suburbs, therefore, consumers residing in lower socioeconomic areas were not represented. The intervention was tested on weekdays, between 9 am and 3 pm, which might have excluded consumers who work during these hours or who visit a community pharmacy on weekends and evening. Due to the nature of feasibility studies, this study had a small sample and is unlikely to be representative of all community pharmacies.

## 5. Conclusions

The proposed interventions and evaluation methods were feasible. The use of posters and badges as situational cues to address barriers to information exchange during OTC consultations was found to be practical in a community pharmacy setting. A larger scale study is now required. There is potential to use situational cues to address other identified barriers to information exchange, to add to the effectiveness of the intervention. With growing emphasis and need for the public to self-care with OTC medicines when appropriate, effective interventions are necessary to promote information exchange, in order to enhance appropriate management in community pharmacies.

## Figures and Tables

**Table 1:** Stages of the intervention.

Week 1	Baseline Data Collection: Audio-Recorded OTC Consultations
Week 2	Pharmacy personnel wore badges Audio-recorded OTC consultations Consumer questionnaire Consumer validation of posters
Week 3	Both posters displayed in the pharmacies No badges worn Audio-recorded OTC consultations
Week 4	Both posters displayed in the pharmacies Badges worn Audio-recorded OTC consultations
Following four weeks	Semi-structured interviews with pharmacy personnel

**Table 2:** Demographic characteristics of consumer participants.

	ProfRole Poster#	InfoExchange Poster*
**Participant, n**		
**Pharmacy 1**	18	19
**Pharmacy 2**	19	19
**TOTAL**	37	38
**Female, n (%)**	27 (73)	28 (74)
**Median age, (IQR) years**	62 (44–71)	61 (48–76)
**Marital status, n (%)**		
**Single**	10 (27)	11 (29)
**Married**	18 (49)	20 (53)
**Other**	9 (24)	7 (18)
**Highest education, n (%)**		
**High school**	10 (27)	11 (29)
**Technical or vocational training**	6 (16)	3 (8)
**University**	18 (49)	19 (50)
**Other**	3 (8)	5 (13)
**Language spoken at home, n (%)**		
**English**	37 (100)	38 (100)
**Usual pharmacy, n (%)**		
**This pharmacy**	22 (59)	28 (74)
**Another pharmacy**	7 (19)	4 (11)
**I don’t have one**	8 (22)	6 (16)

# Poster addressing the professional role of pharmacists.

* Poster modelling information exchange during an OTC consultation.

**Table 3:** Consumer questionnaire responses—before and after poster review.

		ProfRole Poster (n = 37)			InfoExchange Poster (n = 38)		
		Mean (SD)		Mean Change	Mean (SD)		Mean Change
TDF * Domain	Question/Statement ^	Before	After		Before	After	
Knowledge	1. I know that pharmacists are qualified to assist me with my enquiry.	6.46 (0.84)	6.54 (0.56)	0.08	6.55 (0.72)	6.53 (0.65)	–0.02
	2. I know what the pharmacist’s role and responsibilities are.	5.76 (1.46)	5.89 (1.56)	0.13	6.26 (0.98)	6.24 (1.00)	–0.02
	3. I know that giving information to the pharmacist about my health will benefit me.	6.19 (0.97)	6.19 (1.31)	0	6.58 (0.83)	6.58 (0.76)	0
Environ-mental Context	4. I can tell who is a pharmacist and who is a pharmacy assistant.	5.03 (1.82)	5.16 (1.94)	0.13	5.53 (1.64)	5.97 (1.42)	0.44
Social and Professional Role and Identity	5. It is a pharmacist’s job and responsibility to ask me questions about my health.	5.68 (1.72)	6.27 (1.04)	0.59	5.66 (1.63)	6.18 (1.14)	0.52
	6. I trust the pharmacist, as a health professional, to discuss what is best for my enquiry.	6.24 (1.01)	6.41 (0.80)	0.17	6.53 (0.69)	6.66 (0.48)	0.13
	7. It is the duty of the pharmacist, as a health processional, to make sure the medicine they recommend/supply is appropriate for me.	6.19 (1.52)	6.38 (1.01)	0.19	6.50 (0.65)	6.53 (0.76)	0.03
Belief about Capability	8. I am confident that if I want a specific medicine I can provide information about my health with the pharmacist.	6.17 (1.08)	6.33 (0.99)	0.16	6.54 (0.65)	6.59 (0.60)	0.05
	9. I am confident that I can decide if an OTC medicine is appropriate for my condition without help.	4.38 (1.78)	4.43 (2.02)	0.05	4.50 (1.66)	4.19 (1.97)	–0.31
	10. Providing information about my health is (difficult-easy) ^#^	6.08 (0.89)	6.22 (0.95)	0.14	6.50 (0.86)	6.34 (0.91)	–0.16
Belief about Consequences	11. When I provide information about my health, the pharmacist will make sure the medicine is safe and appropriate for me.	6.20 (1.23)	6.23 (1.11)	0.03	6.53 (0.60)	6.50 (0.56)	–0.03
	12. When I provide information about my health, the pharmacist will keep it confidential/private.	6.62 (0.55)	6.70 (0.57)	0.08	6.44 (0.97)	6.56 (0.94)	0.12
	13. When I buy a medicine OTC, I do not need advice as OTC medicines are safe.	3.70 (2.12)	3.89 (2.07)	0.19	3.59 (1.91)	3.68 (2.00)	0.09
Intentions	14. I will definitely provide health information to the pharmacist when purchasing a product/medicine (e.g., ibuprofen, Nurofen^®^).	5.11 (1.98)	5.32 (1.75)	0.21	5.66 (1.85)	5.95 (1.47)	0.29
	15. I intend to provide health information to the pharmacist when I need help with a symptom (e.g., a headache).	5.78 (1.69)	5.86 (1.55)	0.08	6.21 (1.30)	6.47 (0.69)	0.26
	16. How strong is your intention to provide health information to the pharmacist when purchasing a medicine OTC? (not at all strong—very strong) ^^	5.47 (1.54)	5.61 (1.59)	0.14	6.00 (1.19)	6.13 (1.12)	0.13

* TDF: Theoretical Domains Framework. ^ Likert responses varied from 1—‘strongly disagree’ to 7—‘strongly agree’ except ^#^1—‘difficult’ to 7—‘easy’; and ^^ 1—‘not at all strong’ to 7—‘very strong’.

**Table 4:** Demographic information about participating pharmacy personnel.

		Pharmacy 1 (n = 7)		Pharmacy 2 (n = 5)		Total (n = 12)
**Position, n**		Pharmacist, 3	Pharmacy assistant, 4	Pharmacist, 3	Pharmacy assistant, 2	Pharmacy personnel, 12
**Age, median (IQR) years**		51.0 (40–56)	39.5 (33–42)	52.0 (40–55)	25.5 (23–28)	39.5 (29–51)
**Gender, n**	**Female**	3	4	2	2	11
**Employment** **status, n**	**Full time**	1	1	2	1*	5
	**Part-time**	2	2	1	0	5
	**Casual**	0	1	0	0	1
**Pharmacy experience, median (IQR) years**		27.0 (18–34)	11.3 (9–14)	31.0 (20–37)	9.3 (6–13)	14.0 (8–28)

* missing data.

## Supplementary Materials

The following are available online at https://www.mdpi.com/2226-4787/7/2/73/s1. Supplementary Material 1, Supplementary Material 2, Supplementary Material 3, Supplementary Material 4, Supplementary Material 5.

## References

[1] Hibbert D., Bissell P., Ward P.R. (2002). Consumerism and professional work in the community pharmacy. Sociol. Health Illn..

[2] Hughes C.M., McElnay J.C., Fleming G.F. (2001). Benefits and risks of self medication. Drug. Saf..

[3] Bennadi D. (2014). Self–medication: A current challenge. J. Basic. Clin. Pharm..

[4] Blenkinsopp A., Bradley C. (1996). Over the Counter Drugs: Patients, society, and the increase in self medication. BMJ.

[5] Cohen J.P., Paquett C., Cairns C.P. (2005). Switching prescription drugs to over the counter. BMJ.

[6] Therapeutic Goods Administration (2017). Reasons for Scheduling Delegate’s Final Decisions. https://www.tga.gov.au/scheduling-decision-final/scheduling-delegates-final-decisions-june-2017.

[7] Association of the European Self–Medication Industry Switch from Prescription to OTC. http://www.aesgp.eu/facts-figures/otc-ingredients/#undefined.

[8] US Food and Drug Administration Over-the–Counter (OTC) Drug Product Review Process. https://www.fda.gov/Drugs/DevelopmentApprovalProcess/SmallBusinessAssistance/ucm052786.htm.

[9] Watson M.C., Hart J., Johnston M., Bond C.M. (2008). Exploring the supply of non-prescription medicines from community pharmacies in Scotland. Pharm. World Sci..

[10] Dwamena F., Holmes–Rovner M., Gaulden C.M., Jorgenson S., Sadigh G., Sikorskii A., Lewin S., Smith R.C., Coffey J., Olomu A. (2012). Interventions for providers to promote a patient-centred approach in clinical consultations. Cochrane Database Syst. Rev..

[11] Berger K., Eickhoff C., Schulz M. (2005). Counselling quality in community pharmacies: Implementation of the pseudo customer methodology in Germany. J. Clin. Pharm. Ther..

[12] Brata C., Gudka S., Schneider C.R., Everett A., Fisher C., Clifford R.M. (2013). A review of the information–gathering process for the provision of medicines for self–medication via community pharmacies in developing countries. Res. Social Adm. Pharm..

[13] Chee G., Borowitz M., Barraclough A. (2009). Private Sector Health Care in Indonesia.

[14] Alhomoud F., Aljamea Z., Almahasnah R., Alkhalifah K., Basalelah L., Alhomoud F.K. (2017). Self–medication and self–prescription with antibiotics in the Middle East–do they really happen? A systematic review of the prevalence, possible reasons, and outcomes. Int. J. Infect. Dis..

[15] Khalifeh M.M., Moore N.D., Salameh P.R. (2017). Self-medication misuse in the Middle East: A systematic literature review. Pharmacol. Res. Perspect..

[16] U.K Government Medicines Act 1968. http://www.legislation.gov.uk/ukpga/1968/67/introduction.

[17] Government of Western Australia Medicines and Poisons Act 2014. https://www.legislation.wa.gov.au/legislation/statutes.nsf/law_a147008.html.

[18] Therapeutic Goods Administration Poisons standard June 2017. Australian Government Department of Health: Australian Capital Territory 2017. https://www.legislation.gov.au/Details/F2017L00605/Download.

[19] Government of Western Australia Pharmacy Regulations 2010. https://www.slp.wa.gov.au/legislation/statutes.nsf/main_mrtitle_12114_homepage.html.

[20] National Association of Pharmacy Regulatory Authorities NDS Process and Scheduling Factors. https://napra.ca/nds-process-and-scheduling-factors.

[21] New Zealand Government Classification of Medicines. https://www.medsafe.govt.nz/profs/class/clascon.asp.

[22] US Food and Drug Administration OTC (Nonprescription) Drugs. https://www.fda.gov/Drugs/DevelopmentApprovalProcess/HowDrugsareDevelopedandApproved/ucm209647.htm.

[23] US Food and Drug Administration Prescription Drugs and OTC Drugs: Questions and Answers. https://www.fda.gov/drugs/questions-answers/prescription-drugs-and-over-counter-otc-drugs-questions-and-answers.

[24] Association of the European Self–Medication Industry. http://www.aesgp.eu/about-us/who-we-are/.

[25] Watson M., Bond C., Grimshaw J., Johnston M. (2006). Factors predicting the guideline compliant supply (or non-supply) of non–prescription medicines in the community pharmacy setting. Qual. Saf. Health Care.

[26] Watson M.C., Bond C.M., Johnston M., Mearns K. (2006). Using human error theory to explore the supply of nonprescription medicines from community pharmacies. Qual. Saf. Health Care.

[27] Schneider C.R., Everett A.W., Geelhoed E., Kendall P.A., Clifford R.M. (2009). Measuring the assessment and counselling provided with the supply of non-prescription asthma reliever medication: A simulated patient study. Ann. Pharmacother..

[28] Benrimoj S.I., Werner J.B., Raffaele C., Roberts A.S., Costa F.A. (2007). Monitoring quality standards in the provision of non-prescription medicines from Australian Community Pharmacies: Results of a national programme. Qual. Saf. Health Care.

[29] Seubert L.J., Whitelaw K., Boeni F., Hattingh L., Watson M.C., Clifford R.M. (2017). Barriers and facilitators for information exchange during over–the–counter consultations in community pharmacy: A focus group study. Pharmacy.

[30] Medical Research Council Developing and Evaluating Complex Interventions: New Guidance. https://www.mrc.ac.uk/documents/pdf/complex-interventions-guidance.

[31] Rutter P.M., Horsley E., Brown D.T. (2004). Evaluation of community pharmacists’ recommendations to standardized patient scenarios. Ann. Pharmacother..

[32] Watson M.C., Cleland J.A., Bond C.M. (2009). Simulated patient visits with immediate feedback to improve the supply of over-the–counter medicines: A feasibility study. Fam. Pract..

[33] Michie S., Atkins L., West R. (2014). The Behaviour Change Wheel. A Guide to Designing Interventions.

[34] Atkins L., Francis J., Islam R., O’Connor D., Patey A., Ivers N., Foy R., Duncan E.M., Colquhoun H., Grimshaw J.M. (2017). A guide to using the Theoretical Domains Framework of behaviour change to investigate implementation problems. Implement. Sci..

[35] Michie S., Johnston M., Gellman M.D., Turner J.R. (2013). Behavior change techniques. Encyclopedia of Behavioral Medicine.

[36] Seubert L.J., Whitelaw K., Hattingh L., Watson M.C., Clifford R.M. (2018). Interventions to enhance effective communication during over-the–counter consultations in the community pharmacy setting: A systematic review. Res. Social Adm. Pharm..

[37] Seubert L.J., Whitelaw K., Hattingh H.L., Watson M.C., Clifford R.M. (2018). Development of a theory–based intervention to enhance information exchange during Over-The-Counter consultations in community pharmacy. Pharmacy.

[38] Pharmacy Board of Australia Guidelines on Practice–Specific Issues. https://www.pharmacyboard.gov.au/Codes-Guidelines.aspx.

[39] Western Australian Government Pharmacy Act 2010. https://www.slp.wa.gov.au/Index.html.

[40] Michie S., Johnston M., Abraham C., Lawton R., Parker D., Walker A. (2005). Making psychological theory useful for implementing evidence based practice: A consensus approach. Qual. Saf. Health Care.

[41] Michie S., Van Stralen M., West R. (2011). The behaviour change wheel: A new method for characterising and designing behaviour change interventions. Implement. Sci..

[42] West R. (2005). Theory of Addiction.

[43] Steg L., Van Den Berg A.E., De Groot J.I.M. (2013). Environmental Psychology an Introduction.

[44] Bowen D.J., Kreuter M., Spring B., Cofta–Woerpel L., Linnan L., Weiner D., Bakken S., Kaplan C.P., Squiers L., Fabrizio C. (2009). How we design feasibility studies. Am. J. Prev. Med..

[45] Orsmond G.I., Cohn E.S. (2015). The distinctive features of a feasibility study: Objectives and guiding questions. OTJR (Thorofare N J).

[46] Antill J.M., Woodhead R.W. (1990). Critical Path Methods in Construction Practice.

[47] Wujec T. TED: Got a Wicked Problem? First, Tell Me How You Make Toast. https://www.youtube.com/watch?v=_vS_b7cJn2A.

[48] Huijg J.M., Gebhardt W.A., Crone M.R., Dusseldorp E., Presseau J. (2014). Discriminant content validity of a theoretical domains framework questionnaire for use in implementation research. Implement. Sci..

[49] Commonwealth of Australia 2033.0.55.001–Census of Population and Housing: Socio–Economic Indexes for Areas (SEIFA), Australia, 2016. https://www.abs.gov.au/ausstats/abs@.nsf/Lookup/by%20Subject/2033.0.55.001~2016~Main%20Features~FAQs%20-%20SEIFA%202016~4.

[50] Papies E.K. (2017). Situating interventions to bridge the intention–behaviour gap: A framework for recruiting nonconscious processes for behaviour change. Soc. Personal. Psychol. Compass.

[51] Commonwealth of Australia QuickStats 2016 Census. https://www.abs.gov.au/websitedbs/D3310114.nsf/Home/2016%20QuickStats.

[52] Shah B., Chewning B. (2006). Conceptualizing and measuring pharmacist patient communication: A review of published studies. Res. Social Adm. Pharm..

[53] Golafshani N. (2003). Understanding reliability and validity in qualitative research. Qual. Rep..

[54] McMillan S.S., Kelly F., Sav A., King M.A., Whitty J.A., Wheeler A.J. (2014). Consumer and carer views of Australian community pharmacy practice: Awareness, experiences and expectations. J. Pharm. Health Serv. Res..

[55] Malson G. Making Your Pharmacy’s Sales Space as Profitable as Possible. https://www.pharmaceutical-journal.com/opinion/comment/making-your-pharmacys-sales-space-as-profitable-as-possible/10040907.article?firstPass=false.

