# Supplementary material for: A Theory Based Intervention to Enhance Information Exchange during Over-The-Counter Consultations in Community Pharmacy: A Feasibility Study
Source: Pharmacy (Basel). 2019 Jun 20;7(2):73. doi: 10.3390/pharmacy7020073 (PMC6630978; doi:10.3390/pharmacy7020073)
Supplement: Supplementary file 1 [file pharmacy-07-00073-s001.zip › Supplementary Material 1 Posters .pdf]

Supplementary Material 1

Your pharmacist:

- University-qualified health professional
- Health and medicine expert
- Keeps information confidential

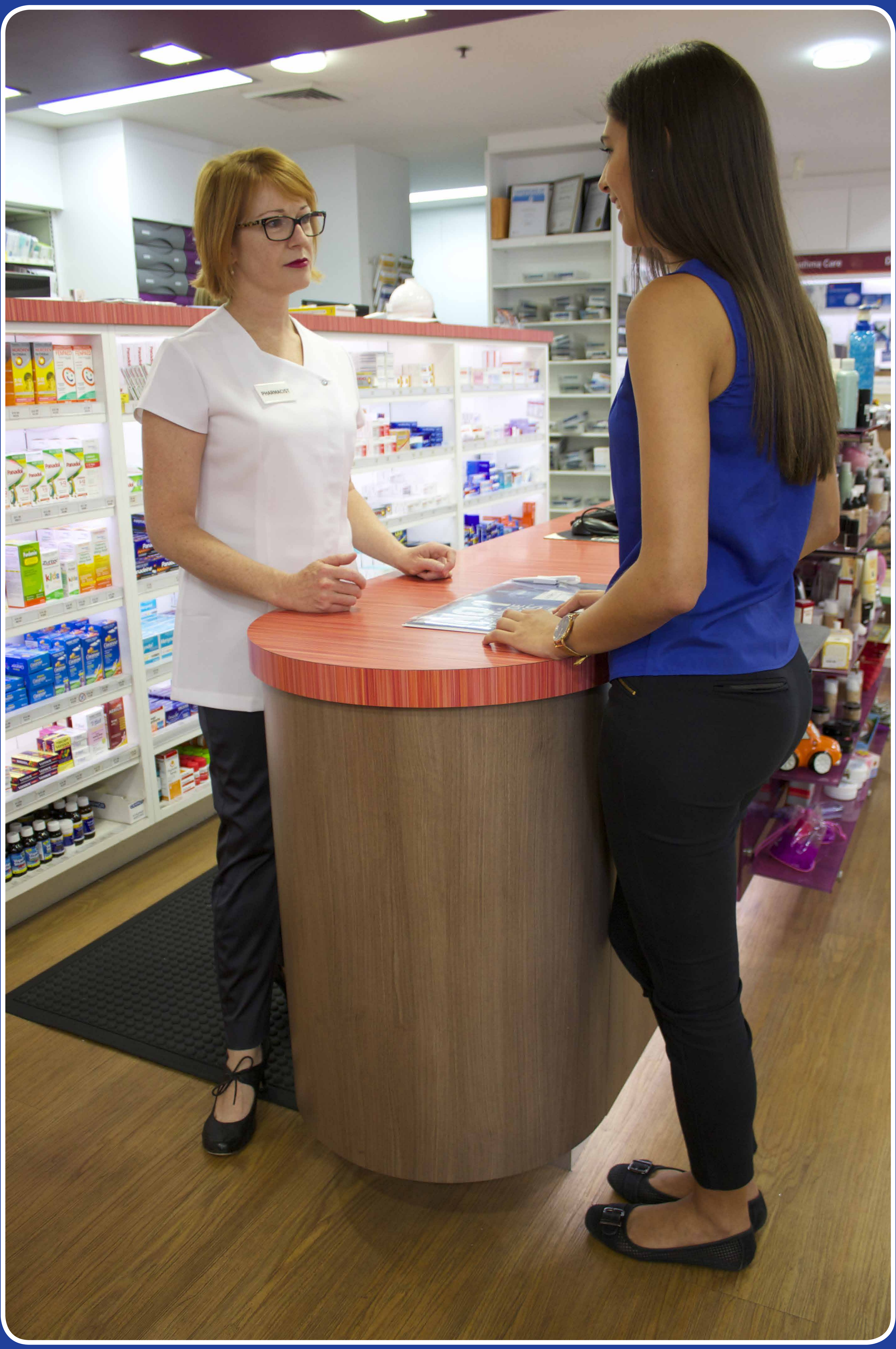

Your pharmacist asks questions to ensure your best health.

THE UNIVERSITY OF WESTERN AUSTRALIA

Curtin University

UNIVERSITY OF ABERDEEN

Need a Medicine?

Talk to your pharmacist.

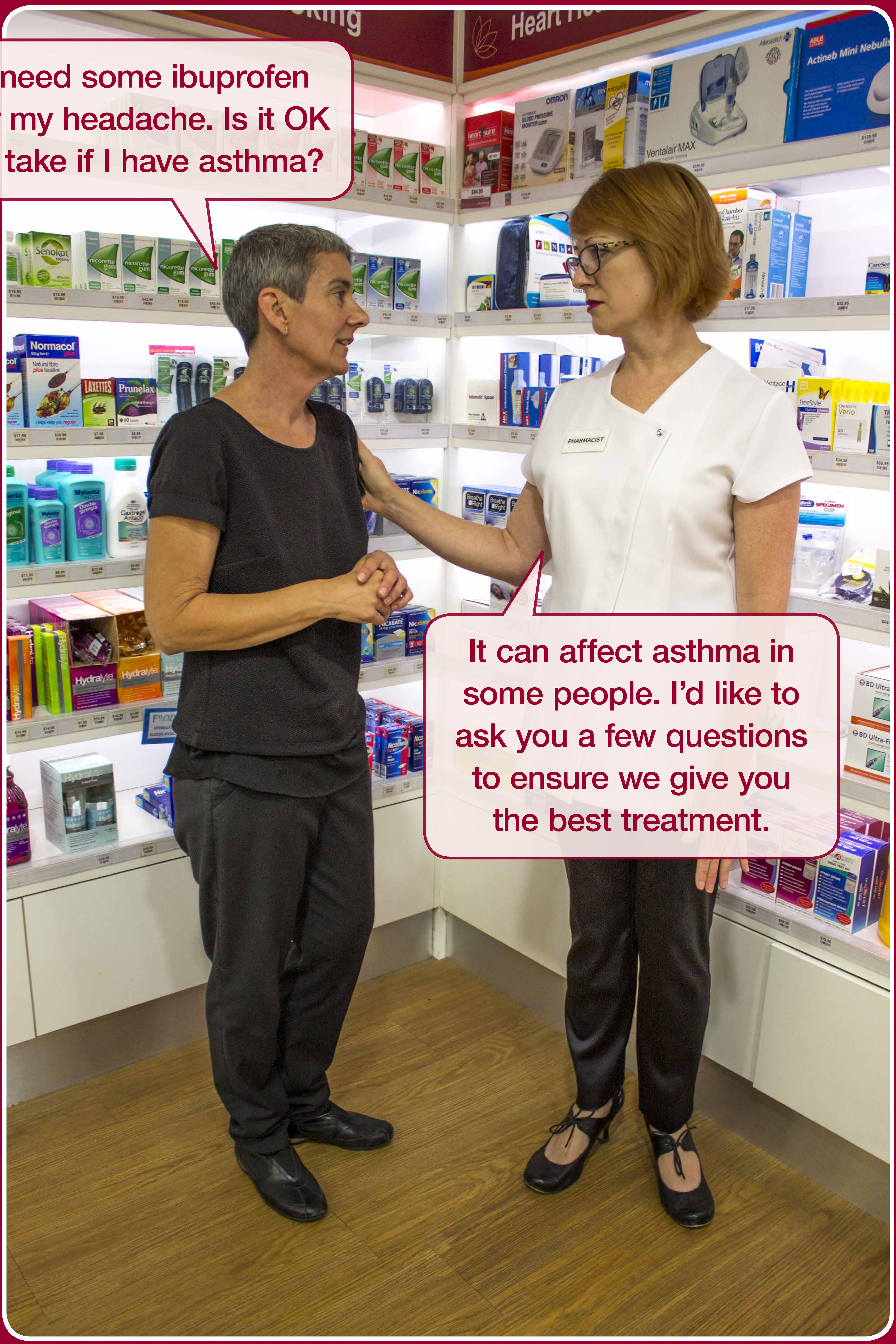

Your pharmacist asks questions for your safety.

UniPrint 130739

THE UNIVERSITY OF WESTERN AUSTRALIA

Curtin University

UNIVERSITY OF ABERDEEN
