# Supplementary material for: A Theory Based Intervention to Enhance Information Exchange during Over-The-Counter Consultations in Community Pharmacy: A Feasibility Study
Source: Pharmacy (Basel). 2019 Jun 20;7(2):73. doi: 10.3390/pharmacy7020073 (PMC6630978; doi:10.3390/pharmacy7020073)
Supplement: Supplementary file 1 [file pharmacy-07-00073-s001.zip › Supplementary Material 2 Badges.pdf]

Supplementary Material 2

PHARMACIST

PHARMACY ASSISTANT
