# Supplementary material for: A Theory Based Intervention to Enhance Information Exchange during Over-The-Counter Consultations in Community Pharmacy: A Feasibility Study
Source: Pharmacy (Basel). 2019 Jun 20;7(2):73. doi: 10.3390/pharmacy7020073 (PMC6630978; doi:10.3390/pharmacy7020073)
Supplement: Supplementary file 1 [file pharmacy-07-00073-s001.zip › Supplementary Material 3 Consumer Questionnaire Before and After.pdf]

Supplementary Material 3

Theoretical Domains Framework domains with the adapted items[1]

When I go to a pharmacy with an "over the counter" (OTC) enquiry….		
TDF Domain*	Item	Likert response
B Cap	providing information about my health is	difficult - easy
B Con	and I buy a medicine OTC, I do not need advice as OTC medicines are safe.	strongly disagree - strongly agree
B Cap	I am confident that if I want a specific medicine I can provide information about my health with the pharmacist.	strongly disagree - strongly agree
B Cap	I am confident that I can decide if an OTC medicine is appropriate for my condition without help.	strongly disagree - strongly agree
B Con	and I provide information about my health, the pharmacist will keep it confidential/private.	strongly disagree - strongly agree
B Con	and I provide information about my health, the pharmacist will make sure the medicine is safe and appropriate for me.	strongly disagree - strongly agree
ECR	I can tell who is a pharmacist and who is a pharmacy assistant.	strongly disagree - strongly agree
ID	it is a pharmacist's job and responsibility to ask me questions about my health.	strongly disagree - strongly agree
ID	it is the duty of the pharmacist, as a health professional, to make sure the medicine they recommend/supply is appropriate for me.	strongly disagree - strongly agree
ID	I trust the pharmacist, as a health professional, to discuss what is best for my enquiry.	strongly disagree - strongly agree
Int	I intend to provide health information to the pharmacist when I need help with a symptom (e.g. a headache).	strongly disagree - strongly agree
Int	How strong is your intention to provide health information to the pharmacist when purchasing a medicine OTC?	not at all strong - very strong
Int	I will definitely provide health information to the pharmacist when purchasing a product/medicine (e.g. ibuprofen, Nurofen®).	strongly disagree - strongly agree
K	I know what the pharmacist's role and responsibilities are.	strongly disagree - strongly agree
K	I know that pharmacists are qualified to assist me with my enquiry.	strongly disagree - strongly agree
K	I know that giving information to the pharmacist about my health will benefit me.	strongly disagree - strongly agree

*K - Knowledge; ECR - Environmental context & resources; ID - Social & Professional role & identity; B Cap - Belief about capability; B Con - Belief about consequences; Int - Intentions

1. Huijg JM, Gebhardt WA, Crone MR, Dusseldorp E, Presseau J. Discriminant content validity of a theoretical domains framework questionnaire for use in implementation research. Implementation Science : IS. 2014;9(1):11-. doi:10.1186/1748-5908-9-11.

General information:

Gender: ☐ Male ☐ Female

Age (years): ____________

Marital Status: ☐ Single ☐ Married
☐ **other:** ______________________

Highest education: ☐ High School ☐ TAFE
☐ University ☐ **other:** _______________

Employment: ☐ Employed ☐ Unemployed
☐ Student ☐ Retired
☐ **other:** __________________

Where were you born? __________________

If you were born outside Australia, what age were you when you came to Australia? __________________

First language: ☐ English ☐ **other:** __________________

Language spoken at home: ☐ English ☐ **other:** _______________

Health Status: ☐ Excellent ☐ Very Good
☐ Good ☐ Poor

My usual pharmacy is:
☐ This pharmacy ☐ Another pharmacy
☐ I don't have one

I visit a pharmacy: ☐ Weekly ☐ Fortnightly
☐ Monthly ☐ Less than monthly

Thank you for your participation.

P:
SitCue:
#

Community Pharmacy Survey

We are conducting a study in community pharmacies and are interested in your thoughts about when you go to a pharmacy with an "over the counter" (OTC) enquiry. That is, when you go to the pharmacy to **ask advice about a symptom** (e.g. headache) or to **buy a medicine** (e.g. paracetamol, *Panadol*®).

We would appreciate your responses to some questions about this. There are no right or wrong answers. Please tell us what you really think.

Some of the questions may *appear to be similar* so please read each question carefully and circle the number that best describes your opinion.

Note: We are **not** asking your thoughts about prescription medicines.

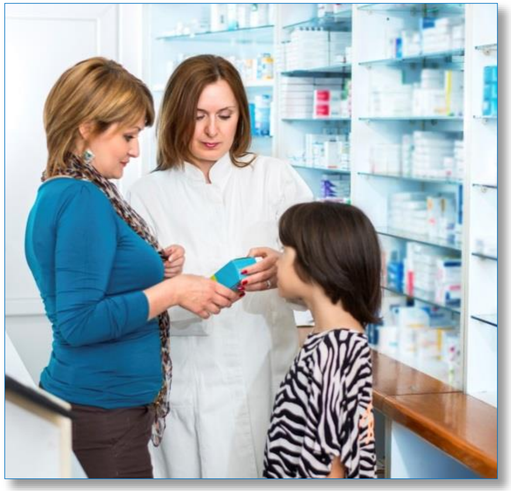

The University of Western Australia   Curtin University   University of Aberdeen

Community Pharmacy Survey

When I go to a pharmacy with an "over the counter" (OTC) enquiry…

I can tell who is a pharmacist and who is a pharmacy assistant.	strongly disagree:_1_:_2_:_3_:_4_:_5_:_6_:_7_:strongly agree
and I provide information about my health, the pharmacist will make sure the medicine is safe and appropriate for me.	strongly disagree:_1_:_2_:_3_:_4_:_5_:_6_:_7_:strongly agree
I know what the pharmacist's role and responsibilities are.	strongly disagree:_1_:_2_:_3_:_4_:_5_:_6_:_7_:strongly agree
I intend to provide health information to the pharmacist when I need help with a symptom (e.g. a headache).	strongly disagree:_1_:_2_:_3_:_4_:_5_:_6_:_7_:strongly agree
it is a pharmacist's job and responsibility to ask me questions about my health.	strongly disagree:_1_:_2_:_3_:_4_:_5_:_6_:_7_:strongly agree
providing information about my health is	difficult:_1_:_2_:_3_:_4_:_5_:_6_:_7_:easy
and I buy a medicine OTC, I do not need advice as OTC medicines are safe.	strongly disagree:_1_:_2_:_3_:_4_:_5_:_6_:_7_:strongly agree
I know that pharmacists are qualified to assist me with my enquiry.	strongly disagree:_1_:_2_:_3_:_4_:_5_:_6_:_7_:strongly agree
and I provide information about my health, the pharmacist will keep it confidential/private.	strongly disagree:_1_:_2_:_3_:_4_:_5_:_6_:_7_:strongly agree
it is the duty of the pharmacist, as a health professional, to make sure the medicine they recommend/supply is appropriate for me.	strongly disagree:_1_:_2_:_3_:_4_:_5_:_6_:_7_:strongly agree
How strong is your intention to provide health information to the pharmacist when purchasing a medicine OTC?	not at all strong:_1_:_2_:_3_:_4_:_5_:_6_:_7_:very strong
I trust the pharmacist, as a health professional, to discuss what is best for my enquiry.	strongly disagree:_1_:_2_:_3_:_4_:_5_:_6_:_7_:strongly agree
I am confident that if I want a specific medicine I can provide information about my health with the pharmacist.	strongly disagree:_1_:_2_:_3_:_4_:_5_:_6_:_7_:strongly agree
I am confident that I can decide if an OTC medicine is appropriate for my condition without help.	strongly disagree:_1_:_2_:_3_:_4_:_5_:_6_:_7_:strongly agree
I will definitely provide health information to the pharmacist when purchasing a product/medicine (e.g. ibuprofen, Nurofen®).	strongly disagree:_1_:_2_:_3_:_4_:_5_:_6_:_7_:strongly agree
I know that giving information to the pharmacist about my health will benefit me.	strongly disagree:_1_:_2_:_3_:_4_:_5_:_6_:_7_:strongly agree

Thank you for your participation.

P:
SitCue:
#

Community Pharmacy Survey

We are conducting a study in community pharmacies and are interested in your thoughts about when you go to a pharmacy with an "over the counter" (OTC) enquiry. That is, when you go to the pharmacy to **ask advice about a symptom** (e.g. headache) or to **buy a medicine** (e.g. paracetamol, *Panadol*®).

We would appreciate your responses to some questions about this. There are no right or wrong answers. Please tell us what you really think.

Some of the questions may *appear to be similar* so please read each question carefully and circle the number that best describes your opinion.

Note: We are **not** asking your thoughts about prescription medicines.

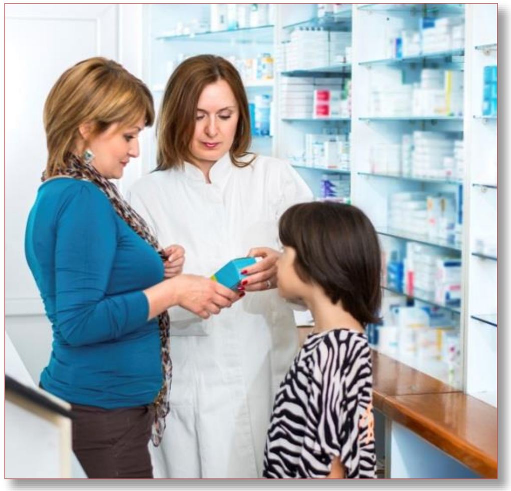

THE UNIVERSITY OF WESTERN AUSTRALIA
Curtin University
UNIVERSITY OF ABERDEEN

Community Pharmacy Survey

When I go to a pharmacy with an "over the counter" (OTC) enquiry…

I can tell who is a pharmacist and who is a pharmacy assistant.	strongly disagree:_1_:_2_:_3_:_4_:_5_:_6_:_7_:strongly agree
and I provide information about my health, the pharmacist will make sure the medicine is safe and appropriate for me.	strongly disagree:_1_:_2_:_3_:_4_:_5_:_6_:_7_:strongly agree
I know what the pharmacist's role and responsibilities are.	strongly disagree:_1_:_2_:_3_:_4_:_5_:_6_:_7_:strongly agree
I intend to provide health information to the pharmacist when I need help with a symptom (e.g. a headache).	strongly disagree:_1_:_2_:_3_:_4_:_5_:_6_:_7_:strongly agree
it is a pharmacist's job and responsibility to ask me questions about my health.	strongly disagree:_1_:_2_:_3_:_4_:_5_:_6_:_7_:strongly agree
providing information about my health is	difficult:_1_:_2_:_3_:_4_:_5_:_6_:_7_:easy
and I buy a medicine OTC, I do not need advice as OTC medicines are safe.	strongly disagree:_1_:_2_:_3_:_4_:_5_:_6_:_7_:strongly agree
I know that pharmacists are qualified to assist me with my enquiry.	strongly disagree:_1_:_2_:_3_:_4_:_5_:_6_:_7_:strongly agree
and I provide information about my health, the pharmacist will keep it confidential/private.	strongly disagree:_1_:_2_:_3_:_4_:_5_:_6_:_7_:strongly agree
it is the duty of the pharmacist, as a health professional, to make sure the medicine they recommend/supply is appropriate for me.	strongly disagree:_1_:_2_:_3_:_4_:_5_:_6_:_7_:strongly agree
How strong is your intention to provide health information to the pharmacist when purchasing a medicine OTC?	not at all strong:_1_:_2_:_3_:_4_:_5_:_6_:_7_:very strong
I trust the pharmacist, as a health professional, to discuss what is best for my enquiry.	strongly disagree:_1_:_2_:_3_:_4_:_5_:_6_:_7_:strongly agree
I am confident that if I want a specific medicine I can provide information about my health with the pharmacist.	strongly disagree:_1_:_2_:_3_:_4_:_5_:_6_:_7_:strongly agree
I am confident that I can decide if an OTC medicine is appropriate for my condition without help.	strongly disagree:_1_:_2_:_3_:_4_:_5_:_6_:_7_:strongly agree
I will definitely provide health information to the pharmacist when purchasing a product/medicine (e.g. ibuprofen, Nurofen®).	strongly disagree:_1_:_2_:_3_:_4_:_5_:_6_:_7_:strongly agree
I know that giving information to the pharmacist about my health will benefit me.	strongly disagree:_1_:_2_:_3_:_4_:_5_:_6_:_7_:strongly agree
