# Supplementary material for: A Theory Based Intervention to Enhance Information Exchange during Over-The-Counter Consultations in Community Pharmacy: A Feasibility Study
Source: Pharmacy (Basel). 2019 Jun 20;7(2):73. doi: 10.3390/pharmacy7020073 (PMC6630978; doi:10.3390/pharmacy7020073)
Supplement: Supplementary file 1 [file pharmacy-07-00073-s001.zip › Supplementary Material 4 Consumer and Pharmacy Personnel interview guides.pdf]

Supplementary Material 4

Semi-structured interview guide for consumers after reviewing a poster.

1. What is the message you get from the poster?
2. When you looked at the poster, what drew your attention first?<br>• Why do you think that?
3. What do you find to be effective in this poster?<br>• Are there particular words that are effective? Why?
4. What do you find is NOT effective in this poster?<br>• Is there something you don't agree with or you find confusing? Explain.
5. Now that we've talked about the poster a little, please sum up what you think the poster means.
6. Do you have any other comments you'd like to make?

Semi-structured interview guide for pharmacy personnel after the intervention.

1. What are your past experiences with OTC queries?
2. Thinking about OTC queries, what changes have you seen in the past few weeks?
3. Experiences with the research process:<br>• How do you feel about having research and researchers in the pharmacy?<br>• What are your thoughts on wearing the recorder?<br>• Do you feel it altered the way you work?<br>• Did you encounter any problems with the research process?<br>• What worked well with the research process?<br>• What would you recommend to improve the process?
