# Supplementary material for: A Theory Based Intervention to Enhance Information Exchange during Over-The-Counter Consultations in Community Pharmacy: A Feasibility Study
Source: Pharmacy (Basel). 2019 Jun 20;7(2):73. doi: 10.3390/pharmacy7020073 (PMC6630978; doi:10.3390/pharmacy7020073)
Supplement: Supplementary file 1 [file pharmacy-07-00073-s001.zip › Supplementary Material 5 Consumer Interview data.pdf]

Supplementary Material 5

Semistructured interview feedback on posters

**Interview number**	1	2	3	4	5	6	7	8	9	10
**Pharmacy**	1	1	1	1	1	1	1	1	1	1
**1 = ProfRole Poster; 2 = InfoExchange Poster**	2	2	2	2	2	2	2	2	2	2
What is the message you get from the poster?	Customer is asking for help and also giving information	Ibuprofen is not appropriate for asthma suffers	informative asking questions about medicaton might be taking	ask your pharmacist before taking any medication	reasonable	pharmacist being caution about medication	the pharmacist is there to give advice and provide answers to questions you don't have the answer to	The pharmacist provides health advice in a pale manner and are qualified to do so	pharmacist helping customer to get the right	That you need to speak to a pharmacist about your sympoms
When you looked at the poster, what drew your attention first?	both people work here	caring look of pharmacist body language	bold writing top and bottom	people and speech bubble	friendly pharmacist	question	TALK	the picture	the pharmacist	talk to your pharmacist
Why do you think that?	regular customer so know the people in photo	emotional connection		stands out again background		because where I looked! What is it trying to tell me	large …	the engagement between the pharmacist and customer	knew the pharmacist --> Amanda is recognisable	the large font
What do you find to be effective in this poster?	nothing	pharmacist looks genuine	bold message at top of poster	speech bubbles	believable		conversational tone of "talk" colour--> conversational	the professionalism of the interaction (white coat)	Amanda is caring for patient	the message that you need to speak to the pharmacist
Are there particular words that are effective? Why?	asthma because I have it and ibuprofen because I won't take it	speech bubbles stand out		talk to your pharmacist - large text		none	"talk" -->conversational	yes, your pharmacist asks questions for your safety	speech bubble	talk to your pharmacist
What do you find is NOT effective in this poster?	pharmacist is in consumers personal	no		no		no	the speech bubbles --> too busy	the speech bubbles are too busy (not clear what the message is)	no	the dialogue bubbles
Is there something you don't agree with or you find confusing? Explain.	no	no		no		no	the hand on the shoulder may come across as patronising especially in younger patients	no	no	too small and wasn't engaging
Now that we've talked about the poster a little, please sum up what you think the poster means.	need for trust between customer and pharmacist	pharmacists should ask questions - asking question will get you better health care	good to ask "deeper" are we on medication	ask your pharmacist about medication if you have any medical conditions	pharmacist asking questions	informative for people who have doubts about pharmacy being able to assist and their medical based knowledge	the pharmacist is here to improve your health	the pharmacist is a health professional that provides high quality service	promoting talking to your pharmacist about medications	the importance of speaking to the pharmacist
Do you have any other comments you'd like to make?	is the poster preaching to the converted - people who look at poster may be already aks questions									

11	12	13	14	15	16	17	18	19	20	21	22	23	24
1	1	1	1	1	1	1	1	1	1	1	1	1	1
2	2	2	2	2	2	2	2	2	1	1	1	1	1
pharmacist making best assessment appropriate to the medication to benefit the consumer	customers should provide info to pharmacist	checking to make sure medicine is correct	need to ask questions to get the best treatment	share medical history with pharmacist	talk to your pharmacist	sharing of information between staff and customer	pharmacist acting responsibly	asking questions	university qualified and they ask questions	what it says	that pharmacists are knowledgeable, confident and there to help	pharmacist provide good customer service	the pharmacist is there to give advice
the words	speech bubble	speech bubbles	the way the pharmacist is holding patients	speech bubbles	speech bubbles	chemist	photo	the people	university qualified	person in uniform	the white uniform	the picture	interaction in picture
wanted to know what info was part of the message	stand out against background	see what it's all about	nice/kind gesture	white background on red	stands out	in line of sight	scene that is expected in pharmacy	drawn to people	very clear to help me reinforce confidence		thinking about the 1st question of survey	"good-looking-bird"	big
body language --> building rapport	conversation in text bubble	display itself	medicines behind them	clear ideas, common questions asked in pharmacy	looks official	encouraging people to talk to pharmacist, pharmacists are approachable and friendly	question asked	pharmacists giving advice	is effective getting message across	not effective because eye drawn to picture only	the interaction between pharm/customer	no comment, nothing in particular	1 on 1 interaction
"I'd like to ask you a few questions" actually getting permission first	talk to your pharmacist; prominent	nope	ask questions --> need to, to get the right treatment	is it ok?; response of pharmacist	talk to your pharmacist; placement at top	is it ok to take; last line - your pharmacist ask questions for your safety	indicated has a medical condition pharmacist reply	talk to your pharmacist		only top box of words	qualified	not particularly	no
not	not very engaging if it was up somewhere, not if I would look at it	nope	nothing	nothing	no	busy, too many items on poster	no	no				dull	the text was too small/ not engaging
no	no	nope	no	no	no	no	no	no	don't like photo	no		no	no, well structured
pharmacists are there to help with medical assessment	telling customers to ask questions of pharmacists to get the best health care	promoting pharmacy as a health representative	confidence in this pharmacy; always there to help	talk to your pharmacist before you get medications	talk to your pharmacist	ask questions before taking medication	using medicine safely	get info from pharmacists	photo displays professionalism, gives confidence, serios and comfortable	did not feel the poster helped of showed her anything no information	a pharmacist is a knowledgeable, confident alternative to a doctor for health advice	pharmacies provide good services	the pharmacist is there to give you professional advice
											Demarked that it was clearer who the pharmacist was after seeing poster		

25	26	27	28	29	30	31	32	33	34	35	36	37	38
1	1	1	1	1	1	1	1	1	1	1	1	1	1
1	1	1	1	1	1	1	1	1	1	1	1	1	1
pharmacist and customer exchanging information	The pharmacist is making sure you're geting the right medicine	The girls enquiring about something and the pharmacist fills her in with detail and info	consultation	someone seeking info	would take the person behind the counter as an assistant	Excluded	pharmacist is giving perso info after asking a question	They're professionally trained health providers	pharmacies wnat pharmacists to do work that would normally be done by a doctor; pharmacies want more business	You should talk to your pharmacist	trust your pharmacist to help you	pharmacist is listening to customer	pharmacist looks helpful
pharmacist white jacket	Amanda	The pharmacist	eye to eye contact between the two	the girls	connecting		pharmacist	Very first line - "University qualified…"	picture	two people talking --> 1 in pharmacist get up I recognise and customer	the people	body language	people
associates white jackets with professionals	been at this pharmacy for 40 years	she seems confident in what's for …	displays interacion	cause I like girls	got a good attitude		that's what they're talking about	Read from top down	natural to look at a picture before you read it	recognised the dress	look at people first naturally	posture and stance, look for people naturally	most obvious thing
in the pharmacy, on site and facing each other	one-on-one, pharmacist interested enough to enquire, personal contact	the outcoming for the young lady from what the pharmacist is advising	pharmacist is th one most visible and open stance consumer talking and discussing	communication	looks as if she's got time		pharmacist looks like she's interested in the customer …	Overall message	pharmacists are professional and trustworthy people	counter with conversation	privacy and confidentiality message; pharmacist looks positive	pharmacist is listening and patient is talking	body language of pharmacist is confident
qualified, expert, confidentiality -->use of language taught us to recognise them as value words	confidential, knowledgeable and expertise, interest - I'm known here	ask questions --> you want the best product	university qualified, expert --> like to know you're talking to someone of this calibre	no	university qualified, keeps info confidential --> very important		ask questions quote, because they need to know; trust pharmacist to give right medication to children	University qualified - trustworthy; Expert; confidential	something I didn't use before, wouldn't usually volunteer any info, so enlightens me to the fact that medical advice is available	no	university qualified; confidential	health and medicine expert	last line - your pharmacist asks questions to ensure your health
no	nothing	nothing	nope	nothing	not very reallistic --> usually busy, should have a private place for counselling		nothing	nope	difficult to answer	no	nothing	too many words	no
no	nothing	no	no	no	no		no	nope	opinion that OTC aren't particularly effective anyway, only prescription that matter	"Ask questions" generally pharmacists are good, but often I go to my doctor to get information as pharmacists don't know everything and dont' recommend the right products	no	no	no
trust your pharmacist	I can trust the service provided (I'm on an awful lot of medications), friendliness --> having a nice conversation	you can depend on this pharmacy	encouraging people to feel free to discuss the status of your health and why you can ask questions	service	I'm here for you		That you can trust your pharmacist; You've got to be forthcoming with information - it's a two-way street	If you go to your pharmacist you get professional, qualified advice	advert for pharmacy	feel confident about pharmacists being health professionals	communication with your pharmacist	reinforcing with customers that pharmacists are there to help	person asking question and pharmacist giving advice
I don't like when pharmacy assistants quiz me because I know more than them (retired nurse)	Extremely satisfied with my pharmacy				Reading the poster definitely changed my perspective			Interesting that there are two women		neat and tidy			

39	40	41	42	43	44	45	46	47	48	49	50	51	52
2	2	2	2	2	2	2	2	2	2	2	2	2	2
2	2	2	2	2	2	2	2	2	2	2	2	2	2
you should give information to your pharmacist even if you only need something minor	the pharmacist will check that the medicine is appropriate for you before giving you anything	confidence in the pharmacist	you can ask a question and get an answer in the right place	check with the pharmacist before buying medicines	ask clearly what you want, tell symptoms, ask their advice	to ask your pharmacist about interactions with other medications	pharmacist is giving full information to the poster	about asthma; take advice from the pharmacist	The pharmacist can provide info on medication and what you can take with them	the pharmacist is there to provide advice	pharmacist is concerned	pharmacists are here to assist in OTC and find the best treatment	pharmacists are knowledgeable and caring
picture	speech bubbles, interaction	clean and well run	the people, then the words	the photo	caring	red	talk to your pharmacist	the woman in the white coat	the large writing	the interaction of ibuprofen and asthma (nurse)	pharmacist is concerned look	talk to your pharmacist	the picture and the touch of the shoulder
largest feature, colours, stands out	to find out who is saying what		they are foremost in the picture	interaction between pharmacist and customer	hand gesture	bright colour	the way it's presented - white on red	health professional; reassuming-hand-sympathetic	size	nursing instinct	eye contact	important message	previously a counseller knows this can be good but also dangerous
large text	interaction between two people	the layout of the chemist is efficient	it encourages people that pharmacists have time to chat and it's important to ask questions	the photo --> pharmacist coat	communication	body language, head tilt, hand touch, not intimidating, female - more relatable	arm on customer shows that she is caring	she looks reassuring	the text	"Talk to your pharmacist" (large)	confident that pharmacist will help you	large writing	wording
talk to your pharmacist	"is it ok?", "best treatment", "ask questions"	university qualified	talk to your pharmacist		nothing in particular	best treatment - hope for the best treatment	words of customer - shows that they should tell the pharmacist of other symptoms	she is wanting to ask a few questions to ensure the best treatment	"talk" (large)	safety	best treatment	"talk"	
speech bubbles - small font specific to the one issue	no	no	contact doesn't look natural, looks staged		not standing at the counter	no	"need a medicine" - didn't see it	no	speech bubbles - too much text	Hand on shoulder! - patronising; speech bubbles are too busy	not over a counter	nothing	Body language of pharmacist
no	no	no	not sure about the arm contact		no	no	no	no		Hand on shoulder; "it's not like she's dying"	conversation bubble is confusing	no	as above
talk to your pharmacist	the pharmacist is there to assist and advice	if you're sick, go to the pharmacist	education poster	Have confidence in seeking advice from your pharmacist about medicine	That you should get advice before taking things	talk to your pharmacist	encouraging people to talk to their pharmacist, be very open	the woman is reassured and she'll get the right treatment	you should ask the pharmacist before buying medicine	you should talk to your pharmacist about your needs if unsure	information about expectations of pharmacists	the pharmacist knows best, make sure you talk to them	I can trust my pharmacist and they will ask quetions to help me
no	customers clothing dull		no	Overall a good poster		nice big writing	good poster	simple, straight forward		Survey (question5) "duty of care" should replace "job and responsibility"	surprised that interaction is in a corner not over the counter		"When I was asked to analyze the poster, I wouldn't have picked up any lack of empathy"

53	54	55	56	57	58	59	60	61	62	63	64	65	66
2	2	2	2	2	2	2	2	2	2	2	2	2	2
2	2	2	2	2	1	1	1	1	1	1	1	1	1
the pharmacist is knowledgeable and there to help. She has the answer to the ladys question	the person is unsure about the effect, but the pharmacist knows and will question the person to ensure the correct medication	you need information from both sides	The pharmacist will check to make sure your medications will work and for interactions	The pharmacist is the best person to ask if you have a medical condition and want to buy an OTC item	Be confident to ask Questions from the pharmacist and expect an answer	Excluded	Pharmacists are qualified, they know what they're talking about and can provide you with the right medication	An expert will help when you go into a pharmacy	Professionalism; trustworthy	Complicated, too much reading	The pharmacist should know all about drug interactions and keep information confidential; trustworthy	Pharmacists are caring, there to help and easy to talk to. Also young!	Trustworthy and knowledgeable
answer from the pharmacist	the consumer	two ladies in photo	teh photo	speech bubbles	Looks very professional; relaxed and happy customer, focussed pharmacist		Your pharmacist	blue	Blue	Blue	One on one	The pharmacist	Pharmacist's facial expression
larger, stands out more	lacking in colour		the hand on shoulder drew attention	eyes drawn to them	their interaction		Specific to me	don't know	Eye-catching	Colour	Personal; pharmacist not distracted; no barriers	Because when I visit the pharmacy, thats who I look for	Could look friendlier
she is getting the information that she needs; reassuring arm	touching - empathetic (but too empathetic for the scenario - headache)	nothing in particular	that pharmacists are a good source of information	no comment	Photo		The colours draw you in the big photo	interaction	Looking at each other in conversation	Pharmacist looks nice	In a pharmacy setting	Not too busy	Customer happy to share information; pharmacist is listening
asthma, headache, "is it ok?" because she's checking it out with the pharmacist	need a medicine		All together gives of a sense of caring		To ensure your best health		your	health and medicine expert	Expert, confidential, professional	Health, pharmacist, confidential	Professional expert; YOUR pharmacist	Best health	Confidential; expert in medicine and health; they ask you questions
blanket question - not enough information in it	too specific; unprofessional shoes, not very unappealing picture but too empathetic for the scenario (headache)	nothing captures you because it's too busy			Not too interesting			logos	No	Pharmacist should be smiling; too much writing; too much in the background	(could have just upper body; not legs)		
no	selling or advertising ibuprofen			"for your safety" --> maybe rephrase "safety of your health"	Too boring			No	No; very easy to understanding	Health and medicine expert			
your pharmacist is reliable, there to help you and give you the best treatment; feel free to ask your pharmacist any questions	getting people to ask for advice of the effect of various drugs; "is it safe?"	Ask for help when you are buying things OTC	pharmacists provide helpful information	Ask the pharmacist to ensure safety of your health	People should trust the pharmacist more, rather than always going to the GP		My pharmacist has qualification and can provide me in the right medicine to be healthy	They want to gain your confidence	That you can trust the pharmacist	If you go to a pharmacist you will get expert…/ professional	Pharmacist is an expert that can be trusted; so you should tell them about your medical conditions	Pharmacists are there to help and know their stuff	Expert; asking information; kept confidential; best treatment
no	medicine should not be capitalised; "Need a medicine?" in block letters; maybe - need help?; full stops are not required	Good message overall (she even was prompted to go and ask the pharmacist a question)			free, no appontments, no wait time				Interesting being part of the survey	Too muddled			

67	68	69	70	71	72	73	74	75	76	77
2	2	2	2	2	2	2	2	2	2	2
1	1	1	1	1	1	1	1	1	1	1
Communication with the staff	Health and medicine expert	Pharmacists will ask questions about your health to help you	You can trust the pharmacist and can see them instead of doctor	well-trained; uni degree in health; they won't gossip about your health	Professional	To instill confidence that you'll get the right information	Caring	University qualified health professional; confidential	Pharmacist is having a conversation	Pharmacists are qualified professionals
The pharmacist	The pharmacist		The questions	Lady in white shirt	Photo	The orange counter	Photo	Words/ information	Faces	Photo
The interaction between them	She looks helpful		Not sure	Looks like a nurse or doctor	They are talking	Sets up a barrier	One is tall and other is short; asking for information	Because they often think that products in pharmacies are useless; commercial	People - real life situation	Centred, drawn to people
You have somebody to talk to without be interrupted	Simplicity - concentration on what it is about	Picture is welcoming	Confidential and freindly look of pharmacist	neat, clean, spacious	Conversation going on	University- qualified health professional	nothing; doesn't grab me	The information / wording at the top	Photo of pharmacist listening to customer	Reminds that pharmacists are more than shopkeepers
Keeps information confidential	Health and medicine expert; keeps information confidential	Qualified	Confidential	Health and medicine expert	Medicine expert --> nust know their products	Keeps information confidential - reassuring	University qualified professional	University qualified; confidential	Bottom line of writing	University qualified, professional, confidential
		Wording - consider rephrasing		Not really	Nothing	Orange counter; body language of people	Doesn't effect me at all		Not very attractive and engaging, no line to draw you	
		"Health and medicine expert" --> may be over the top? Maybe use "professionally qualified to help with general health"						no one is an "expert"		
You can interact with your chemist	You can get good service	Pharmacists are qualified to help you with general health enquiries	Pharmacists are trustworthy; can seek advice from pharmacist instead of doctor	If you want help - you must aks the pharmacist about interactions with other medications	Encouraging you to talk to your pharmacist	Confidence in people seeking advice prior to or after going to a doctor	Encouraging positive interaction with pharmacists	Pharmacists know a lot about health and medicine; all points are true	Promoting pharmacists as part of health care service	Encouraging people to trust and value the pharmacist
This pharmacy is very good! Professional, polite	Always wonderful and helpful here	Intrusive? --> Questions may be intrusive to some customers who know what product they want		? Medicine expert				Concerns about pain relief medications… --> not sure how much he can rely on pharmacists (from own experience)	Interesting that picked two women, also young customers --> most customers in pharmacies are older; can't see customers face, should be holding medication	Dominant figure in photo is consumer not pharmacist, could have better photo; words are good
